# Acute physical-activity related increases in interoceptive ability are not enhanced with simultaneous interoceptive attention

**DOI:** 10.1038/s41598-022-19235-z

**Published:** 2022-09-05

**Authors:** A. Wallman-Jones, E. R. Palser, V. Benzing, M. Schmidt

**Affiliations:** 1 Institute of Sport Science, University of Bern, 145 Bremgartenstrasse, 3012 Bern, Switzerland; 2 Department of Neurology, University of California, San Francisco, CA 94158 USA

**Keywords:** Psychology, Physiology

## Abstract

Interoception, the sense of the internal body, is proposed to support self-regulation, and consequently influence mental health. Researchers have therefore shown interest in improving the ability to accurately monitor internal signals (i.e., interoceptive accuracy, IAcc). Research suggests that cardiac IAcc is modifiable by both manipulations of interoceptive attention (guided attention towards the internal body), and interoceptive exposure (strategically inducing somatic signals e.g., via physical activity). Whilst successful in isolation, it is unclear whether a combined approach (i.e., directing attention towards the internal body when signals are more salient) could elicit greater benefits. In a 2 × 2 within-subject design, 48 healthy adults (*M*_*age*_ = 25.98 ± 4.73 years, 50% female) completed four 20-min conditions varying in both attentional focus (interoceptive vs exteroceptive) and physical activity (active vs rest), with cardiac IAcc measured immediately after. Results revealed a main effect for physical activity (*p* < 0.001), however, there was no effect for attentional focus (*p* = 0.397), and no interaction effect (*p* = 0.797). Differential analyses showed that a higher sporting background increased sensitivity to physical activity-related increases in cardiac IAcc (*p* = 0.031). Findings indicate that (irrespective of attentional focus) moderate-vigorous physical activity-based interventions have the potential to increase cardiac IAcc, with certain individuals potentially benefiting more.

## Introduction

Interoception, defined as the sense of the physiological condition of the body^1^, governs the regulation of all major biological systems e.g., cardiovascular, gastrointestinal, and thermoregulatory. More specifically, it describes the way in which the central nervous system senses, processes, and integrates internal bodily signals, generating mental representations that continuously accommodate novel experiences^2^. This representation of bodily responses occurs across conscious and subconscious levels, predominantly entering our conscious awareness when bodily signals deviate significantly from homeostatic set points^3^.

The ability to accurately track and monitor these changes—interoceptive accuracy (IAcc)—has been suggested to support self-regulation^4,5^. Whilst primarily serving a key role in basic homeostatic regulation, the higher-level significance of IAcc can be seen in its influences on emotion, cognition, and behaviour. Despite the shared goal of maintaining self-regulation prevailing across all interoceptive domains, the cardiovascular system has been a key target of interoceptive research, with particular focus on the heart for pragmatic reasons, such as ease of signal detection and the ability to easily discriminate between individual heartbeats. Further, certain interoceptive mechanisms of emotional dysregulation have been proposed as specific to the cardiac domain, due to the reduced volitional control of the heartbeat in comparison to other systems, such as respiration^6^. As such, cardiac IAcc has been found to predict the efficiency of many socio-emotional self-regulatory processes, such as emotion processing^7,8^, decision making^9,10^, risk taking^11^, and more globally, ones’ sense of self^12^. Correspondingly, atypical cardiac IAcc scores have been implicated in the aetiology of numerous somatic-based mental health conditions, such as panic disorder, anxiety, and depression^13–15^, as well as sensory-based neurodevelopmental conditions, such as autism spectrum conditions^15–17^. Whilst convincing, these findings do not always replicate^18^, which may reflect differences in sample characteristics (e.g., age, symptom severity, intervention history) or methodology (e.g., inclusion criteria, task instructions, analysis decisions). Maintaining methodological control whilst also taking individual differences into consideration is therefore important for comparison across future research.

Despite previous assumptions that cardiac IAcc is a static, trait-like variable, supported by its high-retest reliability^19^, recent research highlights the potential for state-dependent fluctuations, leading researchers to intepret cardiac IAcc as both a trait and state-like variable^20^. Both momentary state-like changes as well as more steady long-term adaptations have been found in responce to both acute^21–24^ and chronic^25,26^ manipulations respectively. Together this has led to an increased research interest in recent years to better understand the malleability of interoceptive processes in the prevention and treatment of mental health conditions^27,28^. Research suggests that cardiac IAcc may be trained using both manipulations of interoceptive attention i.e., directing attention to the internal body, and interoceptive exposure i.e., strategically inducing somatic sensations. Such findings may be explained by the ensuing heightened perception of bodily signals, which are proposed to enter our conscious awareness predominantly when neural monitoring of visceral organs is stimulated through directed attention^27^, or when the body is functioning outside of regular homeostatic set-points^3^.

Previous studies have implemented various acute interoceptive attention manipulations to encourage self-focus and consequently improve IAcc. Such methods include repeating self-focussed words^23^, the presence of self-facing mirrors^24^, or brief-body scans^29,30^. This is further demonstrated in long-term systematic trainings, such as biofeedback of heartbeat timing^31^ or mindfulness-based interventions directing attention towards internal sensations (e.g., heartbeat, breath, and muscle tension)^32^. In explaining such positive effects, it has been suggested that attention-based manipulations reduce engagement of executive control networks and increase engagement of interoceptive networks, centring on the anterior insula cortex^28^. This is supported by functional imaging studies, demonstrating increased activation in insula, somatomotor, and cingulate cortices during interoceptive attention tasks^3^. Taken together, such findings emphasise attentional mechanisms, implying an opportunity to increase attention towards interoceptive signals rather than modulating the strength of the signals themselves.

In contrast to attention-based manipulations, other methods aim to alter interoceptive perceptions by inducing change in the physical state i.e., interoceptive exposure. One of the most naturally occurring manipulations of interoceptive exposure is physical activity, where increasing intensities increase sympathetic activation, eliciting more salient, and thus more noticeable, bodily signals. These states of heightened arousal and increased signal salience are proposed to increase accuracy in perception through both acute and chronic adaptations. As such, previous studies have shown that short bouts of physical activity momentarily increase IAcc when measured immediately after the cessation of exercise^22,33,34^. Furthermore, longer interoceptive interventions with a physical activity component have demonstrated promise that IAcc could be improved through repeated interoceptive exposure, with effects lasting 3-months post intervention^25,26^. These findings are complemented by positive correlations found between IAcc and indices of physical activity and fitness, further supporting the possibility of long-lasting benefits^4,35^. Such effects have been explained by physical activity-related cardiovascular adaptations, which are proposed to act on the periphery to alter visceral-afferent signal transmission, enhancing the salience of the incoming signals, increasing peripheral sensitivity, and narrowing attentional resources^36^. In addition to peripheral adaptations, neuroimaging studies have demonstrated intensity-dependent activation of the insula cortex when blood flow was measured immediately after different bouts of physical activity^37–39^. This intensity-dependent pattern aligns with the Dual-Mode Theory (DMT)^40^, which proposes that increasing intensities initiate a shift of dominant mechanisms from predominantly cognitive processes to strong ascending interoceptive cues, with a crossover of dominance occurring around the point of the anaerobic threshold (AT). Although most findings support physical activity-related increases in IAcc^22,33,34^, recent research disputes this, where a 20-min aerobic manipulation failed to elucidate any benefits when compared to rest^41^. Whilst such findings may raise concerns over the efficacy of physical activity to manipulate IAcc, the use of light-moderate aerobic exercises in these manipulations cannot be compared against more vigorous intensities considering the proposed intensity-dependent shifts in interoceptive processing. It is therefore possible that the sub-maximal intensities used were not sufficient in raising the salience of interoceptive cues enough to produce any acute adaptations. Taken together, despite showing support for the importance of signal salience in bodily perception, contradictory findings also highlight the need for further, more standardised research considering individualised physical activity intensities.

In physical activities involving elements of interoceptive attention (e.g., yoga, Feldenkrais etc.), it is difficult to distinguish the dominant mechanism, where arguments have been made for both attentional (i.e., increased attentional capacity to internal cues) and physiological (i.e., the increased salience of afferent signals) adaptations^42^. Such activities are proposed to rely equally on cognitive and physiological adaptations in influencing interoceptive ability and promoting well-being^43–47^. This potential interaction effect has therefore made it difficult to disentangle this relationship in previous research, where it is unclear whether benefits result from the intensity of the activity, through the increased attention towards the body, or through the interaction of both factors. Further, it is uncertain whether a combined approach could lead to additional benefits compared to interoceptive attention and exposure manipulations in isolation, by directing attention towards the body when signals are more salient and thus more accessible to conscious awareness. Research is therefore needed to understand the mechanisms which lead to increases in IAcc, where a better understanding of the active ingredients could have significant clinical implications in informing the design of physical-activity-based interventions to promote interoceptive processes.

In this present study, therefore, we aimed to systematically disentangle the effects of interoceptive exposure (in the form of physical activity) and interoceptive attention (directing the focus of attention towards bodily signals) manipulations on cardiac IAcc (henceforth, IAcc). Firstly, against the background of empirical evidence^22–24,30,33,34^, we hypothesised that there would be a significant main effect for both manipulations of physical activity and focus of attention. Secondly, through directing attention towards the bodily signals when they are more salient, we predicted that physical activity and attentional focus would have interactive effects on IAcc. Finally, considering known inter-individual variations in IAcc, with several factors previously shown to influence IAcc both at baseline and in response to experimental stimuli (sporting background and fitness^22,33^, gender^48^, chronic stress^49^, and interoceptive ability^41^), we explored potential differential effects of sporting background, physical fitness [$\dot{\mathrm{V}}$O_2peak_], gender, perceived chronic stress, and interoceptive ability (objective and self-reported) on manipulation response.

## Methods

### Design

A 2 × 2 within-subject design was used including manipulations of both focus of attention (interoceptive vs exteroceptive) and physical activity (active vs rest). Participants completed all four conditions: (a) Interoceptive focus with physical activity (PA + IA), (b) Exteroceptive focus with physical activity (PA + EA), (c) Interoceptive focus at rest (REST + IA) and (d) Exteroceptive focus at rest (REST + EA). Sociodemographic variables (age, height, and weight) were measured during the preliminary baseline visit, and a graded maximal exercise test on the cycle ergometer was used to determine both physical fitness as well as the individual intensities to be used in the subsequent experimental conditions. IAcc was measured once during the preliminary baseline visit, as well as immediately after each 20-min manipulation in the experimental conditions. During the experimental conditions, participants filled out a brief questionnaire to assess acute changes in perceptual and affective states, which was completed at pre-test, during (every 5 min—4 instances in total), and immediately after the cessation of each manipulation (post-test). Overall task enjoyment was assessed at one time point per condition once all other testing was completed (post-test—within 2 min of completion). All conditions were counter-balanced to account for potential learning effects.

### Subjects

In total, 48 students (*M*_*age*_ = 25.98 ± 4.73 years; *M*_*BMI*_ = 22.61 ± 2.62, 50% female) volunteered to take part in this study (Table 1). All participants were students at the University of Bern with half majoring in sport science, and the other half majoring in non-sport focused courses. The sample was gender-balanced to account for proposed differences in IAcc between males and females^48^. The experiment was approved by the ethics committee of the University of Bern (Nr. 2019-05-00003) and conducted in accordance with the Declaration of Helsinki. All participants gave written informed consent. To determine sample size, we conducted a simulated power analysis using the SuperPower Shiny app (https://shiny.ieis.tue.nl/anova_power/). We defined a within-subjects 2 $\times$ 2 design with physical activity (present vs absent) and interoceptive attention (present vs absent). We estimated effects based on the available empirical evidence^22–24,30,33,34^and assumed that the PA + IA condition would result in the highest IAcc (*M* = 0.84, ± 0.20); we further expected that PA + EA (*M* = 0.70, ± 0.20) and REST + IA (*M* = 0.66 ± 0.20) conditions would outperform the REST + EA (*M* = 0.66 ± 0.20) condition. To satisfy counterbalancing requirements, we tested the power of *N* = 24 vs. *N* = 48 participants. Using 2000 simulations, results showed that 48 participants (power = 85.5%) would be more favourable than 24 (power = 55.85%) to detect an interaction effect. Please note that these are rough estimations due to the paucity of empirical evidence available.

**Table 1:** Background characteristics of all participants presented as means ± standard deviation.

Variable	Mean ± SD
Age	25.98 ± 4.73
Interoceptive accuracy^a^	0.69 ± 0.22
Interoceptive confidence^a^	5.15 ± 2.20
Resting heartrate	74.02 ± 11.15
Peak oxygen uptake^b^	44.07 ± 8.03
Body mass index^c^	22.56 ± 3.09
Self-report body-awareness^d^	75.12 ± 11.95
Perceived chronic stress^e^	14.16 ± 5.74

Interoceptive accuracy^a^ (IAcc) as measured by Heartbeat Counting Task (HCT—accuracy range: 0–1, and confidence range: 0–10), Peak oxygen uptake^b^ ($\dot{\mathbf{V}}$O_2peak_), Body mass index^c^ (BMI), Self-report body-awareness^d^—Measured using the body-awareness questionnaire (BAQ-range: 18—126), Perceived chronic stress^e^—measured using the perceived stress scale (PSS10-range: 0–40).

### Procedure

**Figure 1:**
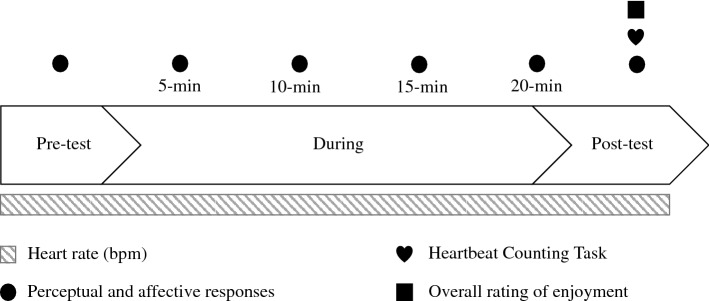
Study design.

Participants were required to visit the laboratory for five separate visits. During the first visit, participants were fitted with heartrate monitoring equipment (mobile heart frequency: Polar Team^2^) on arrival and familiarised with the following procedure. Baseline questionnaires (Body Awareness Questionnaire^50,51^ and Perceived Stress Scale^52,53^) were completed by all participants, and height and weight were measured. Subsequently, interoceptive accuracy and confidence were measured at rest using the Heartbeat Counting Task (HCT), followed by the recording of physiological measures (i.e., resting heartrate, anaerobic threshold, peak power output, $\dot{\mathrm{V}}$O_2peak_, HR_peak_). The initial visit lasted between 45 and 60 min. The four remaining experimental visits lasted approximately 45 min each, in which participants completed each of the four 20-min experimental conditions in a randomised, counterbalanced order. Perceptual and affective responses such as perceived focus of attention^54^, physical/cognitive load^55^, valence/arousal^56^, and acute stress^57^ were measured via questionnaire at six time points (pre-test, during: every 5 min, post-test), whilst overall enjoyment (PACES^58^) and IAcc^59^ were measured once immediately after cessation of the manipulation. Heart rate (bpm) was measured continuously during each condition (Fig. 1). Visits were separated by a minimum of 7 days and a maximum of 10 days and were conducted at the same time of day ± 2 h for each individual participant.

### Experimental manipulations

#### Physical activity manipulation

During the physical activity conditions, incremental intensities were set to ensure that participants surpassed their anaerobic threshold (AT) in the last 5 min (40, 60, 80, 100% AT). These intensities were chosen in line with the DMT^40^, where interoceptive cues are proposed to gain salience with increasing intensities. These intensities were set using the graded maximal exercise test completed at pre-test, where a cycle ergometer was used to assess their aerobic capacity ($\dot{\mathrm{V}}$O_2peak_), anaerobic threshold (AT), peak power output (PPO), and peak heart rate (HR_peak_). During the rest conditions, participants were seated. Identical instructions were given regarding the focus of attention task in both the active and rest conditions.

#### Focus of attention manipulation

For the manipulation of attentional focus, participants listened to an audiotape via headphones that was designed to stimulate either interoceptive or exteroceptive focus of attention. For the interoceptive conditions, the recording instructed participants to focus (non-judgmentally) on internal sensations from different areas of the visceral body (heart, lungs, muscles), similar to previous body-scan interventions^30,60^. The exteroceptive condition was used as a control to ensure that participants focus of attention was directed away from internal bodily sensations, and instead towards external cues. For this, participants listened to a 20-min recording of J.R.R Tolkien’s “The Hobbit” (Der Hörverlag Audiobooks Ltd. 1993). This audiobook has been used successfully as a control condition in previous studies investigating similar themes of internal self-focus and perceptual clarity, where it has been described as occupying the same auditory attention as mindfulness recordings similar to the present study^29,61^. Furthermore, the audiobook was chosen over other experimental auditory stimuli (e.g., white noise) to increase the ecological validity of the manipulation, in which we wanted it to replicate a situation that could occur in real life. Although readers for each recording were matched on gender, it should be noted that different voices were used between the interoceptive and exteroceptive manipulations. Whilst this might be considered to limit comparability, previous studies have found comparable engagement to mindfulness manipulations when using different readers^62^, as well as no significant differences in hyperarousal symptoms, heart rate, or heart rate variability^63^.

### Measures

#### Baseline questionnaires

*Self-reported interoceptive ability* was measured using the Body-Awareness Questionnaire (BAQ)^50^. The BAQ measures attentiveness to normal, non-emotive internal bodily processes and sensations. It is especially sensitive to bodily cycles and rhythms, small changes in normal functioning, and anticipation of bodily reactions. Answers are given on a 7-point Likert scale ranging from 1 (not at all true about me) to 7 (very true about me). Total scores range from 18 to 126, with higher scores indicative of a better body-awareness. The German version of the BAQ has demonstrated both good internal consistency (Cronbach’s α = 0.86) and good construct validity in association with measures of depression and anxiety^52^.

*Chronic stress* was assessed using the German version of the Perceived Stress Scale (PSS-10)^52,53^. This 10-item questionnaire (five-point Likert scale ranging from 0 = “never” to 4 = “very often”) is a validated instrument for assessing chronic perceived stress, measuring the degree to which life in the past month has been experienced as unpredictable, uncontrollable, and overwhelming (e.g., “In the last month, how often have you felt nervous and stressed?”). Total scores range from 0 to 40, with higher scores indicative of a higher level of chronic perceived stress. The German version of the PSS-10 has demonstrated both good internal consistency (Cronbach’s α = 0.84) and good construct validity in association with measures of depression and anxiety^52^.

#### Incremental effort test

Participants completed a graded exercise test on the cycle ergometer to assess their maximal aerobic capacity ($\dot{\mathrm{V}}$O_2peak_), anaerobic threshold (AT), peak power output (PPO) and peak heart rate (HR_peak_). Following a 4-min warm up (Female = 50 W, Male = 100 W), the participants completed a fast ramp protocol with a step wise increase of 25 W min^−1^ with the aim of achieving volitional exhaustion in 8–12 mins^65^. The power output and target HR for each 5-min stage (4 in total) of the active conditions was identified using the VEVO_2_/VECO_2_ slope method from the $\dot{\mathrm{V}}$O_2peak_ data.

#### Affective, perceptual, and physiological responses to the manipulations

*Affective, perceptual, and physiological responses* to the manipulation were recorded to supplement the main findings and elucidate potential mechanistic underpinnings of the effects. This additional information helps to identify any top-down influences on manipulation response, where for example interoceptive focus during exercise has previously been found to produce a higher cognitive strain and lower enjoyment compared to exteroceptive focus^66^.

*Heart rate data* was collected during the entire test session using Polar Team^2^ straps and transmitters (Polar Electro Oy, Kempele, Finland). Mean heart rate during the intervention was used as an objective measure of physiological arousal. During the experimental manipulation, participants completed questionnaires related to their affective state and perceptual response during the intervention.

*Perceived focus of attention* was assessed using a visual analogue scale. Here the participants were asked to answer, “where is your focus of attention right now?”, on a scale ranging from 1 (“Completely internal bodily cues”) to 10 (“Completely external sensory cues”). Although the instrument has not been validated, previous studies using similar methods found associations in expected directions with other variables^54,67,68^.

*Physical and cognitive load* were measured using the 15-point Borg scale^55^, modified to assess for both physical and cognitive strain.

*Valence and arousal* were measured using the self-assessment manikin (SAM)^56^, a non-verbal pictorial assessment technique measuring the pleasure and arousal associated with a person’s affective reaction to a stimulus. The SAM has demonstrated both good internal consistency (Cronbach’s α = 0.88), and acceptable criterion validity^69^.

*Acute stress* was measured using an adapted visual analogue scale asking how stressed the participant felt in that moment, with responses ranging from 1 (“not at all”) to 5 (“very”)^57^. All questionnaires were completed at a total of six time points (pre-test, during: every 5 min, post-test).

*Overall task enjoyment* was assessed at one time point (post-test) using the Physical Activity Enjoyment Scale (PACES)^58^, modified to be suitable for both the active and rest conditions e.g., “During the previous activity…” instead of…” When I am physically active…”. The PACES has previously shown good internal consistency (Cronbach’s α = 0.91)^58^.

### Primary outcome

#### Interoceptive ability

Cardiac interoceptive perception was operationalized as the degree of accuracy (IAcc) in the Heartbeat Counting Task (HCT)^19^. After a practice interval of 20 s, there were three randomised intervals (25, 35, and 45 s) separated by standard resting periods of 20 s^70^. During each interval, participants were given the following instructions: “Without manually checking, can you silently count each heartbeat you feel in your body from the time you hear “start” to when you hear “stop”. Participants were seated throughout the task and were given no information as to the length of the intervals or their performance. In addition, participants were strictly instructed not to guess the number of heartbeats, and to only count those they really felt. The experimenter signalled the beginning and the end of the intervals by announcing “start” and “stop”. Participants were asked to verbally report the number of counted heartbeats straight after the “stop” signal which was electronically recorded by the research team.

Participants’ heartbeats were recorded and analysed using the Polar Team^2^ Pro mobile heart frequency monitor (Polar Electro Oy, Kempele, Finland) which allows for the retrospective analysis of beat-to-beat intervals. Validity and reliability compared to alternative ECG measurement devices has been indicated by previous research^71^. IAcc was computed as the mean heartbeat perception score across the three intervals, calculated according to the following equation:

$$\frac{1}{3}\Sigma 1- \frac{(|n\mathrm{beats } \, \mathrm{real }- n\mathrm{beats}\, \mathrm{reported}|)}{n\mathrm{beats} \, \mathrm{real }}$$

The heartbeat perception score typically takes on values from 0 to 1, where 1 depicts perfect accuracy. Participants’ *confidence* in their response was assessed at the end of each trial using a continuous visual analogue scale (VAS) with verbal descriptors of “Total guess/No heartbeat awareness” and “Complete confidence/Full perception of heartbeat”.

#### Confounding variables

*Prior beliefs about resting heart rate*, their actual *resting heart rate*, *time perception ability*, and *BMI* were measured to account for potential confounds of the Heartbeat Counting Task (HCT)^72–75^. During the time perception control task, as in previous studies^9,70^, participants were required to estimate elapsed time duration for three randomised time intervals (23, 40, 56 s), following the same procedure as the Heartbeat Counting Task (HCT). Bivariate analyses revealed that there were no significant correlations between baseline IAcc and any of the potential confounding variables, including time perception accuracy (*r* = 0.226, *p* = 0.122), prior beliefs about HR (HR estimation accuracy) (*r*_*s*_ = − 0.022, *p* = 0.884), resting HR (*r* = − 0.114, *p* = 0.441), and BMI (*r* = 0.227, *p* = 0.120).

### Statistical analysis

All statistical analyses were conducted using SPSS 27.0 (SPSS Inc., Chicago, IL, USA). All variables were checked for normality using the Shapiro–Wilk test. This indicated that the REST + IA condition was not normally distributed (*p* < 0.05). Following graphical inspection of the data, however, we decided to use parametric tests for the main analyses because of their utility in factorial designs^76^, and the reported robustness of such tests to violations of normality^77^. Further, one missing baseline IAcc value was imputed using the maximum-likelihood EM algorithm method due to an inability of one participant to complete the task correctly.

To assess participant differences and trends in baseline characteristics, bivariate correlations and independent *t-*tests were performed, corrected for multiple comparisons. To check the manipulations were successful and to measure differences in affective and perceptual responses, repeated measures ANOVAs (condition × time) were performed controlling for pre-test values for all variables. For significant findings, Tukey HSD post-hoc analyses were performed to investigate the direction of effects. For the main analyses, two-way repeated measures ANOVAs were used to assess the main and interaction effects between physical activity (active vs rest) and focus of attention (interoceptive vs exteroceptive). To assess for differential effects, ANCOVAs were performed with the chosen factors included as a covariate (i.e., sporting background, physical fitness [$\dot{\mathrm{V}}$O_2peak_], gender, perceived stress, interoceptive ability [objective and subjective]). For *t*-tests, Cohen’s *d* effect sizes were interpreted as small = 0.20, moderate = 0.50, and large = 0.80 (Cohen, 1988). For ANOVA and ANCOVA, partial eta^2^ (ƞ^2^_*p*_), effect sizes were interpreted as small = 0.01, moderate = 0.06, and large = 0.14^78^. Statistical significance was set a priori at *p* < 0.05. For data visualisation, raincloud plots^79^ were created in R Project (R Core 2017).

## Results

### Affective, perceptual, and physiological responses to the manipulations

#### Heart rate

Average heart rate (HR; beats per minute) for each condition was as follows: Physical activity + interoceptive focus [PA + IA] = 134.68 ± 11.37, Physical activity + exteroceptive focus [PA + EA] = 134.29 ± 10.48, Rest + interoceptive focus [REST + IA] = 70.42 ± 8.87, Rest + exteroceptive focus [REST + EA] = 70.12 ± 8.58. Results revealed a significant main effect of condition on heartrate, *F*(3,188) = 674.139, *p* =  < 0.001, *ƞ*_p_^2^ = 0.915. Post-hoc comparisons found no significant difference between the two active conditions (*p* = 0.997), and no significant difference between the rest conditions (*p* = 0.999). In both active conditions, however, heartrate was significantly higher than the rest conditions (*ps* < 0.001).

#### Focus of attention

**Figure 2:**
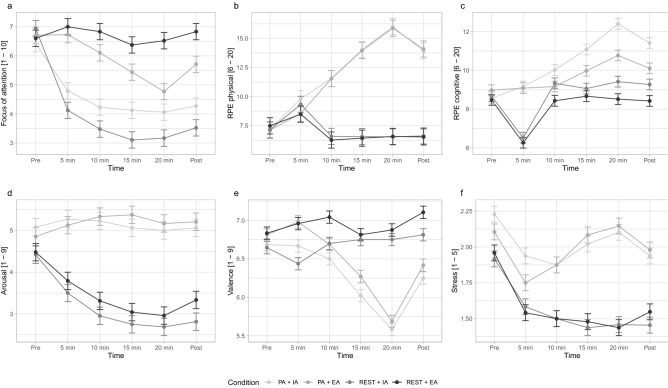
Line graphs depicting the affective and perceptual responses to each condition, recorded before (pre), during (5, 10, 15, 20 min), and after (post) the manipulation. (**a**) Focus of attention; measured using a VAS scale with lower scores indicating internal focus, and higher scores indicating external focus. (**b**) RPE physical; measured using the Borg scale, with higher numbers indicative of increased physical strain. (**c**) RPE cognitive; measured using an adapted Borg scale, with higher number indicative of increased cognitive strain. (**d**) Arousal; measured using the Self-Assessment Manikin, with higher scores indicative of increased arousal. (**e**) Valence; measured using the Self-Assessment Manikin, with higher scores indicating positive valence, and lower scores indicating negative valence. (**f**) Stress; as measured using a VAS scale, with higher scores indicating higher states of acute stress. Error bars shown standard error, and scales are displayed on the Y-axis.

A repeated measures ANOVA revealed a significant effect of condition *F*(3,187) = 47.179, *p* =  < 0.001, *ƞ*_p_^2^ = 0.401, no significant effect of time, *F*(3,552) = 1.920, *p* = 0.105, *ƞ*_p_^2^ = 0.010, and a significant interaction between time and condition, *F*(9,552) = 3.479, *p* =  < 0.001, *ƞ*_p_^2^ = 0.053 on attentional focus (Fig. 2). Post-hoc comparisons revealed internal focus to be significantly high in the REST + IA condition compared to the REST + EA condition (*p* ≤ 0.001). Similarly, internal focus was rated as significantly higher in the PA + IA condition compared to the PA + EA condition (*p* ≤ 0.001). In addition, PA + IA was significantly higher than both rest conditions; REST + IA (*p* = 0.035) and REST + EA (*p* =  < 0.001), which was emulated in the PA + EA condition; REST + IA (*p* ≤ 0.001) and REST + EA (*p* = 0.008) (see Fig. 2a & Appendix 1).

#### Rating of perceived exertion (RPE)-physical

A repeated-measures ANOVA revealed a significant effect of condition *F*(3,187) = 358.035, *p* =  < 0.001, *ƞ*_p_^2^ = 0.852, a significant effect of time, *F*(2,391) = 48.317, *p* =  < 0.001, *ƞ*_p_^2^ = 0.205, and a significant interaction between time and condition, *F*(6,391) = 100.120, *p* =  < 0.001, *ƞ*_p_^2^ = 0.616 perceived exertion (Fig. 2). Post-hoc comparisons found RPE-*physical* scores to be significantly higher during the PA + IA condition compared to both rest conditions (*ps* ≤ 0.001). Similarly, RPE-*physical* scores were significantly higher during the PA + EA condition compared to both rest conditions (*ps* ≤ 0.001). There were no significant differences between rest conditions (*p* = 0.919) or active conditions (*p* = 0.752) (see Fig. 2b & Appendix 1).

#### Rating of perceived exertion (RPE)-cognitive

A repeated-measures ANOVA revealed a significant effect of condition *F*(3,187) = 18.892, *p* =  < 0.001, *ƞ*_p_^2^ = 0.233, a significant effect of time, *F*(3,474) = 50.980, *p* =  < 0.001, *ƞ*_p_^2^ = 0.214, and a significant interaction between time and condition, *F*(8,980) = 6.804, *p* =  < 0.001, *ƞ*_p_^2^ = 0.098 perceived cognition exertion (Fig. 2). Post-hoc comparisons found RPE*cognitive* scores to be significantly higher during the PA + IA condition compared to PA + EA (*p* = 0.050), REST + IA (*p* < 0.001), and REST + EA (*p* < 0.001). Further, RPE-*cognitive* scores were significantly higher during the PA + EA condition compared to both rest conditions: REST + IA (*p* = 0.024), REST + EA (*p* < 0.001). There was no significant difference between the rest conditions (*p* = 0.470) (see Fig. 2c & Appendix 1).

#### Self-assessment manikin (SAM)-arousal

A repeated-measures ANOVA revealed a significant effect of condition *F*(3,187) = 44.071, *p* =  < 0.001, *ƞ*_p_^2^ = 0.414, a significant effect of time, *F*(3,485) = 10.004, *p* =  < 0.001, *ƞ*_p_^2^ = 0.051, and a significant interaction between time and condition, *F*(8,485) = 4.456, *p* =  < 0.001, *ƞ*_p_^2^ = 0.067 perceived arousal (Fig. 2). Post-hoc comparisons found arousal during the PA + IA condition to be significantly higher than both rest conditions (*ps* < 0.001). Similarly, arousal during the PA + EA condition was significantly higher than both rest conditions (*ps* < 0.001). There was no significant difference in arousal between the rest conditions (*p* = 0.575) or the active conditions (*p* = 0.973) (see Fig. 2d & Appendix 1).

#### Self-assessment manikin (SAM)-valence

A repeated-measures ANOVA revealed a significant effect for condition *F*(3,187) = 7.379, *p* =  < 0.001, *ƞ*_p_^2^ = 0.106, a significant effect for time, *F*(3,527) = 50.980, *p* = 0.009, *ƞ*_p_^2^ = 0.018, and a significant interaction between time and condition, *F*(8,527) = 9.193, *p* =  < 0.001, *ƞ*_p_^2^ = 0.129 on perceived valence (Fig. 2). Post-hoc comparisons found pleasure during the PA + IA condition to be significantly lower than the REST + EA condition (*p* = 0.004), whereas there was no significant difference between PA + EA and both rest conditions, REST + IA (*p* = 0.581) and REST + EA (*p* = 0.065). Additionally, there was no significant difference between the rest conditions (*p* = 0.619), the active conditions (*p* = 0.792), or the interoceptive attention conditions (*p* = 0.128) (see Fig. 2e & Appendix 1).

#### Acute stress

A repeated-measures ANOVA revealed a significant effect of condition *F*(3,187) = 8.191, *p* =  < 0.001, *ƞ*_p_^2^ = 0.116, a significant effect of time, *F*(3,593) = 8.102, *p* =  < 0.001, *ƞ*_p_^2^ = 0.042, and a significant interaction between time and condition, *F*(9,567) = 4.428, *p* =  < 0.001, *ƞ*_p_^2^ = 0.033 on acute stress. Post-hoc comparisons found acute stress during the PA + IA condition to be significantly higher than both rest conditions (*ps* = *0.0*02). Acute stress was also significantly higher in the PA + EA compared to the rest conditions: REST + IA (*p* = 0.002), REST + EA (*p* = 0.003). There were no significant differences between the rest conditions or the active conditions (*ps* = 1.00) (see Fig. 2f & Appendix 1).

#### Overall task enjoyment (PACES score after each condition)

A one-way ANOVA for overall enjoyment revealed a significant effect of condition on overall task enjoyment, *F*(3,188) = 7.405, *p* < 0.001, *ƞ*_p_^2^ = 0.106. Post-hoc comparisons found no significant differences between the active conditions (*p* = 0.510), the interoceptive attention conditions (p = 0.966), or the exteroceptive attention conditions (*p* = 0.074). Additionally, there were no significant differences between REST + IA (*M* = 17.02 ± 6.12) and both active conditions: PA + IA (*M* = 16.50 ± 5.52, *p* = 0.966), PA + EA (*M* = 14.95 ± 5.76, *p* = 0.252). The REST + EA condition [*M* = 12.25 ± 4.22], however, was rated as significantly more enjoyable than both interoceptive attention conditions (*ps* < 0.001). Lower scores were indicative of greater enjoyment.

### Main and interaction effects

A two-way repeated measures ANOVA (physical activity vs focus of attention) revealed a significant main effect of physical activity on interoceptive accuracy (IAcc), *F*(1,44) = 14.623, *p* =  < 0.001, *ƞ*_p_^2^ = 0.249. There was, however, no significant main effect of focus of attention, *F*(1,44) = 0.732, *p* = 0.397, *ƞ*_p_^2^ = 0.016, and no interaction between physical activity and focus of attention, *F*(1,44) = 0.067, *p* = 0.797, *ƞ*_p_^2^ = 0.002 (see Table 2 & Appendix 2 and 3).

**Table 2:** Means and standard deviation scores for the interoceptive accuracy and interoceptive confidence scores across all four conditions.

	PA + IA	PA + EA	REST + IA	REST + EA
Interoceptive accuracy	0.81 ± 0.18	0.80 ± 0.17	0.72 ± 0.21	0.70 ± 0.22
Interoceptive confidence	6.63 ± 2.06	6.46 ± 2.33	5.03 ± 2.57	4.79 ± 2.43

*PA + EA* physical activity with an exteroceptive focus, *PA + IA* physical activity with an interoceptive focus, *REST + EA* exteroceptive focus at rest, *REST + IA* interoceptive focus at rest.

The pattern of results observed for IAcc was emulated in the confidence ratings, where there was a main effect of physical activity (*F*(1,44) = 31.525, *p* =  < 0.001, *ƞ*_p_^2^ = 0.417), yet no effect of focus of attention (*F*(1,44) = 1.199, *p* = 0.279, *ƞ*_p_^2^ = 0.027), and no interaction effect between physical activity and focus of attention (*F*(1,44) = 0.035, *p* = 0.852, *ƞ*_p_^2^ = 0.001) (Table 1).

### Differential analyses

**Figure 3:**
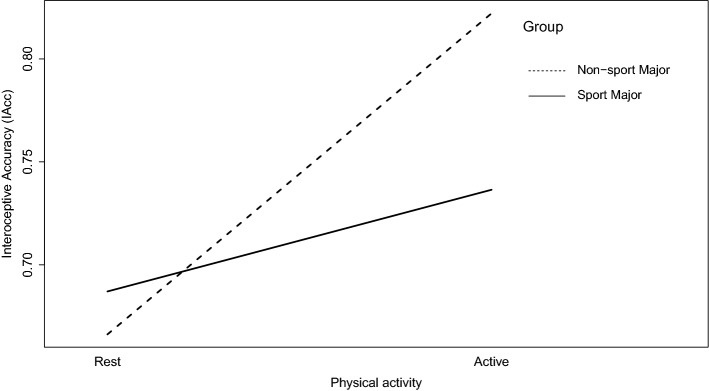
Interaction plot displaying the difference in interoceptive accuracy (IAcc) scores between active and rest conditions, with participants grouped by sporting background (students majoring in sport [*N* = 24] vs non-sport focussed courses [*N* = 24]).

Differential analyses revealed a significant interaction between sporting background (Sport Major vs Non-sport Major) physical activity (active vs rest), *F*(1,46) = 4.944, *p* = 0.031, *ƞ*_p_^2^ = 0.097, however there was no interaction between sporting background and focus of attention (interoceptive vs exteroceptive), *F*(1,46) = 0.017, *p* = 0.898, *ƞ*_p_^2^ = 0.000. The pattern observed for IAcc was emulated by the confidence ratings, where there was a significant interaction between physical activity (active vs rest) and sporting background, *F*(1,46) = 6.343, *p* = 0.015, *ƞ*_p_^2^ = 0.121 (Fig. 3, Table 3).

**Table 3:** Means and standard deviations for interoceptive accuracy (IAcc) and interoceptive confidence scores at baseline and across all four conditions, with participants split by sporting background (students majoring in sport vs non-sport focussed courses).

	Condition				
	Baseline	PA + IA	PA + EA	REST + IA	REST + EA
**Sport Major**					
Interoceptive accuracy	0.74 ± 0.20	0.79 ± 0.19	0.80 ± 0.19	0.77 ± 0.12	0.74 ± 0.18
Interoceptive confidence	5.37 ± 2.29	6.24 ± 2.08	6.21 ± 2.58	5.29 ± 2.54	5.49 ± 2.31
**Non-sport Major**					
Interoceptive accuracy	0.65 ± 0.24	0.83 ± 0.17	0.81 ± 0.16	0.67 ± 0.27	0.66 ± 0.25
Interoceptive confidence	4.94 ± 2.14	7.02 ± 2.00	6.71 ± 2.10	4.78 ± 2.63	4.08 ± 2.39

Independent *t*-tests revealed that there was a trend for $\dot{\mathrm{V}}$O_2peak_ scores to be higher in the Sport Major group, indicated by a medium to large effect size, however there were no differences in age, baseline IAcc and confidence, resting HR, BMI, self-report body-awareness (BAQ) or perceived stress (PSS10) (Table 4). In contrast, however, there were no significant interaction effects for physical fitness ($\dot{\mathrm{V}}$O_2peak_), as well as no interaction with age, baseline IAcc and confidence, resting HR, BMI, self-report body-awareness (BAQ), perceived stress (PSS10), or gender, with either of the two main factors (physical activity and focus of attention) (*ps* < 0.05).

**Table 4:** Background characteristics of participants grouped by sporting background (students majoring in sport vs non-sport focussed courses).

	Non-sport Major Mean ± SD	Sport Major Mean ± SD	*p*	*d*
*N*	24 (50% female)	24 (50% female)		
Age	24.96 ± 3.70	26.96 ± 5.51	> 0.999	0.43
Interoceptive accuracy^a^	0.65 ± 0.24	0.74 ± 0.20	> 0.999	0.41
Interoceptive confidence^a^	4.94 ± 2.14	5.37 ± 2.29	> 0.999	0.19
Resting heartrate	76.29 ± 13.72	71.75 ± 7.43	> 0.999	0.41
Peak oxygen uptake^b^	41.55 ± 7.83	46.59 ± 7.58	0.224	0.65
Body mass index^c^	22.56 ± 3.09	22.67 ± 2.12	> 0.999	0.04
Self-report body-awareness^d^	75.19 ± 11.93	75.04 ± 12.22	> 0.999	0.01
Perceived chronic stress^e^	15.70 ± 6.33	12.63 ± 4.73	0.540	0.55

Data are presented as means ± standard deviations with p-values corrected for multiple comparisons. Interoceptive accuracy^a^ (IAcc) as measured by Heartbeat Counting Task (HCT-accuracy range: 0–1, and confidence range: 0–10), Peak oxygen uptake^b^ ($\dot{\mathrm{V}}$O_2peak_), Body mass index^c^ (BMI), Self-report body-awareness^d^—Measured using the body-awareness questionnaire (BAQ-range: 18–126), Perceived chronic stress^e^—measured using the perceived stress scale (PSS10-range: 0–40).

## Discussion

In this study, we examined the main and interaction effects of acute interoceptive attention (through directed attention towards bodily signals) and interoceptive exposure (through physical activity) on cardiac IAcc. We found that IAcc was significantly higher after physical activity compared to the rest conditions, however, there was no main effect for the focus of attention manipulation, and no interaction between physical activity and focus of attention. In addition, differential analyses found a significant interaction between physical activity and sporting background (i.e., students majoring in sport vs non-sport focussed courses), where individuals with a lower sporting background appeared to benefit the most from the physical activity manipulation. We did not, however, find significant influences of physical fitness ($\dot{\mathrm{V}}$O_2peak_), gender, perceived chronic stress, or baseline interoceptive ability (objective and self-reported). Taken together, our results support and extend previous findings demonstrating the ability for acute physical activity to increase IAcc, whilst also suggesting that no additional benefits are accrued through directing attention towards bodily signals at moderate-vigorous physical intensities.

Although physical activity has already been shown to influence IAcc^22,33,34^, many studies are outdated, underpowered, and did not control for intensities of activity in a standardised and individualised manner. In addition, contradictory findings were recently found, where an acute bout of sub-maximal aerobic physical activity failed to elicit significant increases in IAcc^41^. In the present study, therefore, we accounted for these issues by setting individual intensities from a baseline maximal intensity fitness test. In addition, participants were balanced on variables previously found to contribute to interoceptive variation (gender^48^ and sporting background^22^), and further, other known relevant baseline characteristics (physical fitness^33^, chronic stress^49^, and baseline interoceptive profiles^41^) were taken into consideration through differential analyses. In doing so, our findings support and extend previous findings in emphasising that physical activity of a certain intensity does influence IAcc, however, effects appear to be confined to certain populations.

Whilst we did not find any significant influence of physical fitness, gender, chronic stress, or baseline interoceptive ability, we did find a significant influence of sporting background (i.e., students majoring in sport vs non-sport focussed courses). It has previously been reported that sporting background predicts IAcc both at rest and following physical activity^22^. In line with this, mainly the students majoring in non-sport focussed courses were able to benefit from the physical activity manipulation in the present study. Baseline characteristics provide one explanation for our findings, where the Sport Major group showed a trend towards having higher $\dot{\mathrm{V}}$O_2peak_ scores (*d* = 0.65). Higher fitness levels in the students majoring in sport-focussed courses may have caused differing autonomic responses to the 20-min cycling manipulation. Despite a trend towards significance, however, we found no differential effects for $\dot{\mathrm{V}}$O_2peak_ between the experimental conditions across the whole sample (*ƞ*_p_^2^ = 0.072), and no differences in both resting HR and the maximum HR in the active conditions between both sporting background groups. Future research should measure other indices of autonomic regulation that might differ amongst fitness levels, such as heartrate recovery, where a faster deceleration of HR in those with greater fitness could influence performance in the subsequent Heartbeat Counting Task (HCT) task^80^. The randomised nature of the task should however mitigate any effects of heartrate deceleration with scores calculated by averaging across the different trials.

Aside from physical fitness, we also saw a tendency for differing baseline interoceptive profiles between groups, where the students majoring in non-sport focussed courses displayed a trend for having lower IAcc scores at baseline (Table 4) (*d* = 0.41). This is in line with previous research, demonstrating the ability for baseline IAcc to predict manipulation response^41^ and positive associations between physical fitness and IAcc. Yet in contrast, differential analyses failed to reveal an interaction between physical activity and baseline IAcc across the whole sample. Despite the insignificant output, however, it should be noted that the results displayed a trend towards significance (*ƞ*_p_^2^ = 0.077), therefore the role of baseline IAcc in manipulation response cannot be ruled out. Furthermore, it should be noted that the current study was adequately powered to detect the main and interaction effects of physical activity and interoceptive attention, but not for differential effects analyses. Taken together, whilst our non-sport focussed group do not necessarily represent a physically inactive population, these results hold promise that those most in need can benefit the most from the physical activity manipulation, considering their lower baseline IAcc and the known relationship between sedentary behaviour and poor mental and physical health^81^. Whilst more research is needed to further elaborate on these differential findings, it appears that individual differences in baseline characteristics should be taken into consideration in determining the efficacy of physical activity-based interoceptive interventions.

Previous studies have reported benefits from adding elements of physical activity to interoceptive attention interventions^25,82^, whilst similarly, mindful physical activities (e.g., Yoga and Feldenkrais) have been found to elicit a positive influence on interoceptive outcomes^43,46,47^. To the best of our knowledge, however, this study was the first to systematically explore the potential interaction between attentional focus and physical activity. In contrast to our hypotheses, however, we did not find an interaction between manipulations of physical activity and focus of attention. Whilst we cannot completely rule out the effects of top-down processes from this result, where exercising undoubtedly leads to a natural increased awareness of physiological perturbations irrespective of directed attention. Further, increased expectation of signals elicited through increased monitoring during physical activity may also influence perception^83^. Such findings do indicate, however, that the salience of the signals themselves might be the dominant mechanism at play, rather than increased attentional resources, as alternatively hypothesised. Effects of physical intensity, baseline IAcc, and the timing and delivery of the attentional focus should, however, also be considered in explaining the lack of interaction effect before strong conclusions can be drawn.

The results support that the decision to set the physical intensities to reach and surpass the AT worked in achieving the desired main effect of physical activity, however, this may have overridden potential additional benefits of the interoceptive attention manipulation. In other words, whilst sympathetic activation increases under physical load, so does internal noise (e.g., due to rhythmic movements, elevated breathing rate, etc.)^84^. It is therefore possible that with increasing intensity, the dominance of interoceptive cues diminished available attentional resources for the interoceptive attention task. This was emulated by the significantly higher cognitive strain (RPE-*cognitive*) reported during the physical activity with an interoceptive focus (PA + IA) condition compared to the other conditions, indicating that the interoceptive attention task and the heightened internal noise are competing for attentional resources. If true, this implies that sub-maximal intensities could be more effective for this combined approach, allowing the space for this potential interaction effect to surface. Disputing this hypothesis, a recent study with healthy adults found no improvements in IAcc following an acute (20-min, average HR = 117.43 bpm) bout of sub-maximal yoga^41^. Whilst the average HR was only slightly lower than the active conditions of this present study, the incremental design of our manipulation implies that the maximum HR reached in the final stage was likely to be significantly higher. These null findings therefore oppose the hypothesis that intensity accounts for the lack of interaction in the present study.

A recent pilot study with healthy adults contrastingly found interoceptive benefits using a single bout of sub-maximal yoga of a longer duration (75-min; average HR not significantly different to at rest)^85^. Such differences in effects in comparison to previous yoga manipulations could indicate the need for longer sustained attention for interaction effects to manifest, as hypothesised by the authors. It should be noted, however, that this variance could have also arisen due to the different baseline IAcc scores across both studies (Demartini et al., 2021 = 0.55, Schillings et al., 2021 = 0.65 [yoga]), where lower initial scores leave more room for improvement. Further, the average HR during the yoga classes appear to be significantly lower in this longer yoga session, suggesting benefits are likely to arise predominately from the internal focus component of the manipulation rather than any change in physical intensity. Taken together, these sparse and contradictory findings support the need for further research investigating physical activity characteristics and their effect on IAcc to fully understand the interplay of interoceptive attention and interoceptive exposure at sub-maximal intensities.

In this present study, it appears that the power of effects from the physical activity manipulation have led to the attentional focus manipulation being the least potent ingredient, therefore increasing the intensity of the attentional focus manipulation could be considered. Alternative attentional strategies e.g., biofeedback^21,86^ might provide a stronger stimulus to match the strength of the physical activity stimulus. Alternatively, instead of increasing intensity, the timing of the attentional focus manipulation could be investigated in future studies. For example, a recent intervention delivering an interoceptive attention task (the Heartbeat Counting Task) after the cessation of a short bout of exercise, once the heartrate had returned to resting state, was found effective in improving interoceptive processes in adults with autism spectrum conditions^25^. Utilising such a design would ensure that the bodily signals remain salient from the preceding activity, yet the individual is in a relaxed state, freeing up attentional resources to focus on the attention task. Given their promising results, future research should therefore investigate whether the time-point of the interoceptive attention manipulation (before, after, simultaneous) interacts with the intensity level of the interoceptive exposure manipulation.

In interpreting our findings, some limitations must be considered. Firstly, in contrast to research supporting the ability of acute attention-based manipulations to influence IAcc, we failed to find a main effect for the focus of attention manipulation. Whilst this was likely driven by the overriding salience of the physical activity conditions, further information could be gained by looking at the ratings of enjoyment, where participants rated the REST + IA condition significantly lower than the REST + EA condition. This apparent dislike of the interoceptive task could have affected its efficacy, where the negative affect experienced during the manipulation could have disturbed performance in the subsequent Heartbeat Counting Task (HCT). This is in line with previous research, demonstrating how change in positive affect during experimental manipulations influences subsequent IAcc performance^41^. Contrastingly, this could equally be explained by a greater liking of the exteroceptive focus condition. Whilst there was no significant difference in pleasure reported during the manipulations between the rest conditions (*p* = 0.619), this hypothesis is supported by the higher overall enjoyment of the task reported after the REST + EA condition compared to the REST + IA (*p* < 0.001). Aside from enjoyment, it should also be considered that the audiobook does not represent a true equivalent to the task demands of the interoceptive task. Despite there being no significant difference in perceived cognitive exertion between the rest conditions, there was a trend for higher cognitive exertion in the PA + IA compared to the PA + EA condition (*p* = 0.050). This implies that once accompanied by additional task demands from the physical activity component, the cognitive load of the interoceptive attention task is perceived to be significantly higher than the audiobook. Although alternative exteroceptive methods were considered, e.g., focussing on white noise^87^, the audiobook was selected to be mindful of the 20-min duration of the manipulation, with the goal of having a higher success rate in maintaining attention. This difference in attentional manipulations, however, along with the different readers used, may prevent true comparisons between the manipulations. Future research should therefore look at using alternative attention manipulations that might provide a better comparison to the demands and likability of the interoceptive task.

Secondly, although the Heartbeat Counting Task (HCT) is a widely used research instrument to measure interoceptive processing, the quantification and significance of IAcc measured in this way remains in debate within the field of interoceptive research, with recent reviews failing to replicate previous associations to indices of mental health^18^. Whilst our findings begin to disentangle the mechanisms which lead to increases in IAcc as measured by the Heartbeat Counting Task (HCT), more research is needed to fully understand these processes and their relation to self-regulation and mental health. Further, based on the current discussion concerning the reliability and validity of the Heartbeat Counting Task (HCT)^72,73,80,88^, future research might also use alternative strategies to assess IAcc^89^. This should be taken into consideration in the interpretation of our findings, where IAcc in our study might not reflect a pure representation of cardiac interoception. Nonetheless, for the current study, a short duration test was necessary to monitor acute changes induced by physiological arousal, therefore the Heartbeat Counting Task (HCT) presented a suitable solution to fit the methodology. Future work should therefore look at developing and using alternative methods that have the potential to accurately capture continuous changes in bodily perception during physiological arousal.

Finally, it should be noted that despite progress in the field, we are still far away from fully understanding what an optimal interoceptive profile should look like, and further, how this could differ across clinical and non-clinical populations. In previous studies, for example, an IAcc score of 0.70 and above is generally considered as having a good heartbeat perception^64^. In this case, however, we must also consider whether there could be an instance of having ‘too much of a good thing’, and the dangers that could come from elevating IAcc above this arbitrary threshold, such maladaptive, heightened attention to bodily signals. Nonetheless, research indicates that these risks are more likely in the interoceptive attention domain^15,90^, and thus improvements in precision are likely to lead to a healthier regulation of attention, resulting from a decreased need to attend to ambiguous bodily signals. If true this supports the need for biofeedback elements in interventions, as opposed to primarily attention-based manipulations e.g., mindfulness. Further research is required to test these hypotheses, establishing the potential benefits and risks in elevating IAcc, and how this could differ across populations. On this note, it should also be considered that the baseline IAcc scores were generally high across the whole sample, mostly driven by the sport-major group. Whilst these values are not unexpected in a healthy, athletic population, this does mean that the sample might not be generalisable. This also created a reduced variability and scope for improvement, possibly masking the potential effects of the manipulation. Further research is therefore needed in different age groups, clinical populations, and fitness levels to establish how demographics influence sensitivity to intervention.

This study supports and expands on previous findings demonstrating the ability of acute moderate-vigorous physical activity to increase IAcc. Further, it highlights the importance of taking individual differences into consideration, where individuals who are less involved in sport (students taking a non-sport focussed major) seem to benefit more from the physical activity manipulation than those with a greater sporting background (students taking a sport major). Although further research is needed to advance physical-activity based interventions, it can be concluded from the results that simultaneous interoceptive attention does not seem to contribute additional value to moderate-vigorous physical activity in modulating IAcc. To achieve additional benefits from a combined interoceptive attention manipulation, it may be advisable for physical activities at this threshold to either manipulate interoceptive attention after the cessation of activity, or to increase the potency of the attentional focus manipulation itself. In addition, due to the paucity of empirical evidence, it remains unclear how the interplay between physical activity and IAcc plays out both at lower intensities, as well as in clinical populations. Further research is therefore needed to test these hypotheses and fully understand these interacting mechanisms.

## Supplementary Information

Supplementary Information.

## Data Availability

The datasets generated during and/or analysed during the current study are available from the corresponding author on reasonable request.
