# Supplementary material for: Acute physical-activity related increases in interoceptive ability are not enhanced with simultaneous interoceptive attention
Source: Sci Rep. 2022 Sep 5;12:15054. doi: 10.1038/s41598-022-19235-z (PMC9445090; doi:10.1038/s41598-022-19235-z)
Supplement: Supplementary file 1 — Supplementary Information. [file 41598_2022_19235_MOESM1_ESM.docx]

Acute physical-activity related increases in interoceptive ability are not enhanced with simultaneous interoceptive attention

Wallman-Jones. A^1^*, Palser, E. R.^2^, Benzing. V^1^, Schmidt. M^1^.

^1^Institute of Sport Science, University of Bern, Bern, Switzerland

﻿^2^Department of Neurology, University of California, San Francisco, CA 94158, USA

*Corresponding author: [amie.wallman-jones@unibe.ch](mailto:amie.wallman-jones@unibe.ch),

145 Bremgartenstrasse, 3012 Bern, Switzerland.

| **Variable** | **Condition** | **Pre-test** | **5-min** | **10-min** | **15-min** | **20-min** | **Post-test** |
| --- | --- | --- | --- | --- | --- | --- | --- |
| Focus of attention | PA + IA | 6.42 ± 1.88 | 4.79 ± 1.56 | 4.23 ± 1.60 | 4.12 ± 1.81 | 4.06 ± 2.10 | 4.27 ± 1.65 |
|  | PA + EA | 6.71 ± 1.56 | 6.73 ± 1.44 | 6.10 ± 1.55 | 5.44 ± 1.80 | 4.77 ± 1.89 | 5.71 ± 1.68 |
|  | REST + IA | 6.94 ± 1.76 | 4.13 ± 1.55 | 3.48 ± 1.66 | 3.10 ± 1.65 | 3.17 ± 1.64 | 3.52 ± 1.49 |
|  | REST + EA | 6.60 ± 1.67 | 7.00 ± 1.58 | 6.83 ± 1.73 | 6.38 ± 1.99 | 6.52 ± 1.88 | 6.83 ± 1.58 |
| RPE-physical | PA + IA | 7.17 ± 1.84 | 9.81 ± 2.96 | 11.56 ± 1.54 | 14.02 ± 1.54 | 16 ± 1.66 | 13.92 ± 1.94 |
|  | PA + EA | 7.10 ± 1.89 | 8.56 ± 2.04 | 11.54 ± 1.87 | 13.96 ± 1.61 | 15.87 ± 1.67 | 14.08 ± 2.16 |
|  | REST + IA | 7.15 ± 1.77 | 9.33 ± 2.60 | 6.58 ± 1.13 | 6.56 ± 1.24 | 6.54 ± 1.24 | 6.63 ± 1.23 |
|  | REST + EA | 7.49 ± 2.43 | 8.49 ± 2.23 | 6.27 ± 0.71 | 6.43 ± 0.93 | 6.57 ± 1.32 | 6.53 ± 1.05 |
| RPE-cognitive | PA + IA | 8.56 ± 3.30 | 9.17 ± 1.89 | 10.02 ± 2.07 | 11.08 ± 2.26 | 12.4 ± 3.17 | 11.42 ± 2.70 |
|  | PA + EA | 8.98 ± 3.56 | 9.08 ± 1.71 | 9.17 ± 2.26 | 9.98 ± 2.47 | 10.77 ± 2.87 | 10.10 ± 2.53 |
|  | REST + IA | 8.67 ± 3.28 | 6.52 ± 1.03 | 9.33 ± 2.48 | 9.06 ± 2.13 | 9.42 ± 2.40 | 9.27 ± 2.08 |
|  | REST + EA | 8.48 ± 2.96 | 6.26 ± 0.78 | 8.43 ± 2.24 | 8.67 ± 2.57 | 8.52 ± 2.35 | 8.42 ± 2.28 |
| Arousal | PA + IA | 5.08 ± 1.49 | 5.27 ± 1.38 | 5.23 ± 1.39 | 5.06 ± 1.55 | 5.00 ± 1.80 | 5.06 ± 1.51 |
|  | PA + EA | 4.85 ± 1.57 | 5.13 ± 1.59 | 5.33 ± 1.24 | 5.38 ± 1.48 | 5.17 ± 1.66 | 5.21 ± 1.50 |
|  | REST + IA | 4.42 ± 1.83 | 3.50 ± 1.50 | 2.95 ± 1.48 | 2.75 ± 1.47 | 2.69 ± 1.49 | 2.81 ± 1.36 |
|  | REST + EA | 4.48 ± 1.81 | 3.79 ± 1.57 | 3.31 ± 1.45 | 3.04 ± 1.44 | 2.96 ± 1.49 | 3.33 ± 1.58 |
| Valence | PA + IA | 6.69 ± 1.23 | 6.67 ± 1.10 | 6.50 ± 1.01 | 6.02 ± 1.18 | 5.58 ± 1.44 | 6.25 ± 1.30 |
|  | PA + EA | 6.81 ± 0.84 | 6.98 ± 0.84 | 6.69 ± 0.97 | 6.27 ± 1.27 | 5.69 ± 1.46 | 6.42 ± 1.25 |
|  | REST + IA | 6.65 ± 1.50 | 6.44 ± 1.60 | 6.70 ± 1.28 | 6.75 ± 1.31 | 6.75 ± 1.21 | 6.81 ± 1.35 |
|  | REST + EA | 6.83 ± 1.49 | 6.96 ± 1.32 | 7.04 ± 1.07 | 6.81 ± 1.10 | 6.88 ± 1.21 | 7.10 ± 1.21 |
| Acute stress | PA + IA | 5.08 ± 1.49 | 5.27 ± 1.38 | 5.23 ± 1.39 | 5.06 ± 1.55 | 5.00 ± 1.80 | 5.06 ± 1.51 |
|  | PA + EA | 4.85 ± 1.57 | 5.13 ± 1.59 | 5.33 ± 1.24 | 5.38 ± 1.48 | 5.17 ± 1.66 | 5.21 ± 1.50 |
|  | REST + IA | 4.42 ± 1.83 | 3.50 ± 1.50 | 2.95 ± 1.48 | 2.75 ± 1.47 | 2.69 ± 1.49 | 2.81 ± 1.36 |
|  | REST + EA | 4.48 ± 1.81 | 3.79 ± 1.57 | 3.31 ± 1.45 | 3.04 ± 1.44 | 2.96 ± 1.49 | 3.33 ± 1.58 |

**Appendix 1.** A table of affective and perceptual measures taken at pre-test, during (every 5-minutes), and post-test. Data displayed as mean ± standard deviation.

**Appendix 2.** Raincloud plots showing interoceptive accuracy (IAcc) for all participants across the four conditions. PA + EA: physical activity with an exteroceptive focus, PA + IA: physical activity with an interoceptive focus, REST + EA: exteroceptive focus at rest, REST + IA: interoceptive focus at rest. Error bars display standard error of the mean and boxplots display central tendencies of the data.

**Appendix 3.** Interaction plot displaying the 2x2 interaction effects between physical activity (active vs rest) and focus of attention (interoceptive vs exteroceptive) on interoceptive accuracy (IAcc). Results are displayed as means.
